# Two Cases of Imperforate Hymen—Successful Division of the Membrane

**Published:** 1840-03-07

**Authors:** William Zollickoffer


### TWO CASES OF IMPERFORATE HYMEN—*Successful division of the Membrane.* By William Zollickoffer, M. D.

To the Editors of the Medical Examiner.

Gentlemen,—If you think the inclosed cases worthy of a place in the "Medical Examiner," you are at liberty to give them an insertion. I have made them as short as the nature of the case would admit of.

Very respectfully,

Wm. Zollickoffer.

*Middleburg*, (*Md.*,) 24th Feb., 1840.

*Case* 1.—In July, 1836, I was requested, by Mr. H., to see his little daughter. On my arrival at his residence, Mrs. H. said—"We sent for you to get your opinion in the case of our daughter, who has been in a strange way ever since her birth. She has great difficulty in passing her water, and I think it is owing to her being grown up." I examined the situation of the child, and found a substance occupying the place of the hymen, that resembled in its general appearance the *cutis vera*, through which there was an opening, scarcely large enough to admit of the passage of an ordinary darning needle. I considered it necessary to divide the substance, not only with a view to obviate the existing inconvenience, but to prevent any unpleasant circumstances taking place, should the little girl be permitted to live to the period of menstruation. I gave this as my opinion to the parents. I was requested to operate; and, on the 26th of the above month, after placing her in a suitable position, I divided the membrane, which offered as much resistance to the edge of the scalpel as the skin of a child of three years old. This was the age of this patient. I directed the ungt. simplex to be put on a small tent, and introduced morning and evening, to facilitate the formation of healthy granulations, and to prevent adhesion of the parts. This patient has laboured under no inconvenience since, and no unpleasant consequences grew out of the operation.

In January, 1837, I visited the daughter of Mr. S. From what the mother stated, I was of the opinion that it was a case of imperforate hymen: an examination of the patient confirmed me in the sentiment. No mucous membrane could be seen. In its place there was a substance resembling in its aspect the true skin. I suggested an operation, to which the parents gave their consent; and, before I left the house, I divided successfully the substance which occupied the place of the hymen. The little girl, four years and two months of age, was soon relieved by adopting the treatment directed in the above case. I will here remark, that the above cases were not the result of the want of due care on the part of the parents, from a want of which, excoriations, and consequent adhesion of the labia, sometimes occur. There was *no* adhesion of the parts, but a substance exhibited, displaying the true characteristics of the *cutis vera*.

These are the only cases of *imperforate hymen* that claimed my notice, *in any way*, during a practice of nineteen years previous to the time of my seeing them.

---

*Rigby's Obstetric Memoranda, by* S. C. Foster, M. D.—A useful little vade mecum, embodying much valuable practical information, has just been issued under this title. The practitioner of midwifery will find it a convenient pocket manual.

